# Crystal structure of ethyl 4-(2-chloro­phen­yl)-2-methyl-4*H*-pyrimido[2,1-*b*][1,3]benzo­thia­zole-3-carboxyl­ate

**DOI:** 10.1107/S2056989015014905

**Published:** 2015-08-22

**Authors:** Balbir Kumar, Manmeet Kour, Satya Paul, Rajni Kant, Vivek K. Gupta

**Affiliations:** a Post-Graduate Department of Physics & Electronics, University of Jammu, Jammu Tawi 180 006, India; b Department of Chemistry, University of Jammu, Jammu Tawi 180 006, India

**Keywords:** crystal structure, pyrimido[2,1-*b*][1,3]benzo­thia­zole, ester, biological activity

## Abstract

In the title compound, C_20_H_17_ClN_2_O_2_S, the dihedral angle between the planes of the benzo­thia­zole fused ring system (r.m.s. deviation = 0.024 Å) and the chloro­benzene ring is 89.62 (12)°. The ester C—O—C—C side chain has an *anti* orientation [torsion angle = −155.2 (3)°]. In the crystal, weak aromatic π–π stacking inter­actions are observed between the phenyl and pyrimidine rings [centroid–centroid seperation = 3.666 (2) Å].

## Related literature   

For biological activities of benzo­thia­zoles, see: Landreau *et al.* (2002 ▸); Russo *et al.* (1985 ▸). For a related structure, see: Sankar *et al.* (2015 ▸).
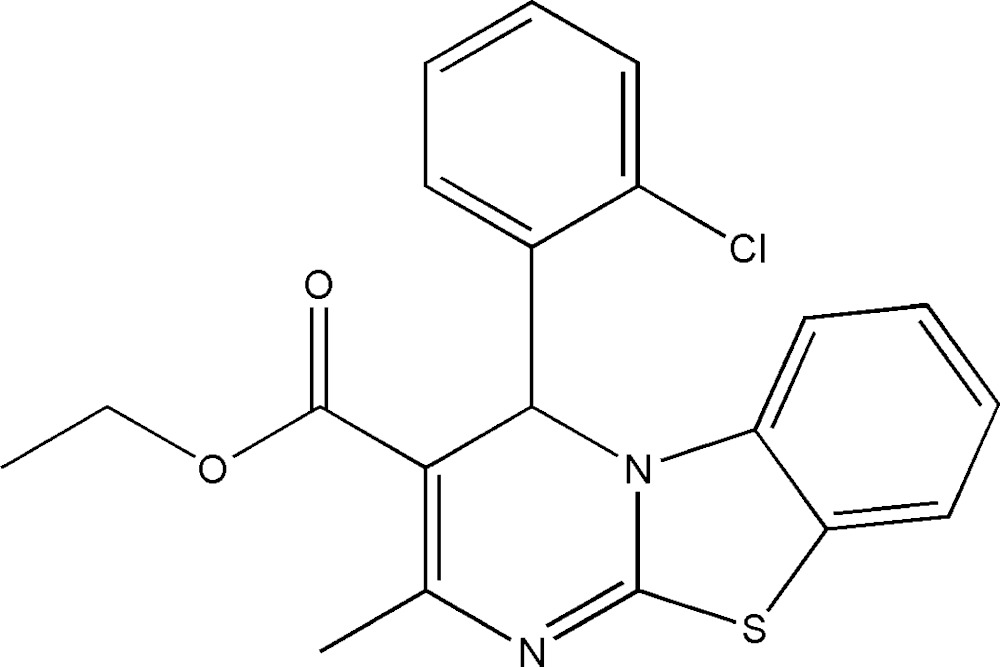


## Experimental   

### Crystal data  

C_20_H_17_ClN_2_O_2_S
*M* *_r_* = 384.87
Triclinic, 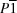

*a* = 8.9049 (8) Å
*b* = 8.9275 (10) Å
*c* = 12.3564 (11) Å
α = 88.434 (8)°
β = 83.536 (7)°
γ = 66.201 (10)°
*V* = 892.90 (15) Å^3^
*Z* = 2
Mo *K*α radiation
μ = 0.35 mm^−1^
*T* = 293 K
0.30 × 0.20 × 0.20 mm

### Data collection  

Oxford Diffraction Xcalibur Sapphire3 diffractometer
Absorption correction: multi-scan (*CrysAlis PRO*; Oxford Diffraction, 2010 ▸) *T*_min_ = 0.880, *T*_max_ = 1.000
6440 measured reflections
3477 independent reflections
2275 reflections with *I* > 2σ(*I*)
*R*_int_ = 0.033

### Refinement  

*R*[*F* ^2^ > 2σ(*F* ^2^)] = 0.053
*wR*(*F* ^2^) = 0.142
*S* = 1.03
3477 reflections
237 parameters
H-atom parameters constrained
Δρ_max_ = 0.64 e Å^−3^
Δρ_min_ = −0.35 e Å^−3^

### 

Data collection: *CrysAlis PRO* (Oxford Diffraction, 2010 ▸); cell refinement: *CrysAlis PRO*; data reduction: *CrysAlis PRO*; program(s) used to solve structure: *SHELXS97* (Sheldrick, 2008 ▸); program(s) used to refine structure: *SHELXL97* (Sheldrick, 2008 ▸); molecular graphics: *ORTEP-3 for Windows* (Farrugia, 2012 ▸); software used to prepare material for publication: *PLATON* (Spek, 2009 ▸).

## Supplementary Material

Crystal structure: contains datablock(s) I, New_Global_Publ_Block. DOI: 10.1107/S2056989015014905/hb7475sup1.cif

Structure factors: contains datablock(s) I. DOI: 10.1107/S2056989015014905/hb7475Isup2.hkl

Supporting information file. DOI: 10.1107/S2056989015014905/hb7475Isup3.cml

**Fig. 1.**
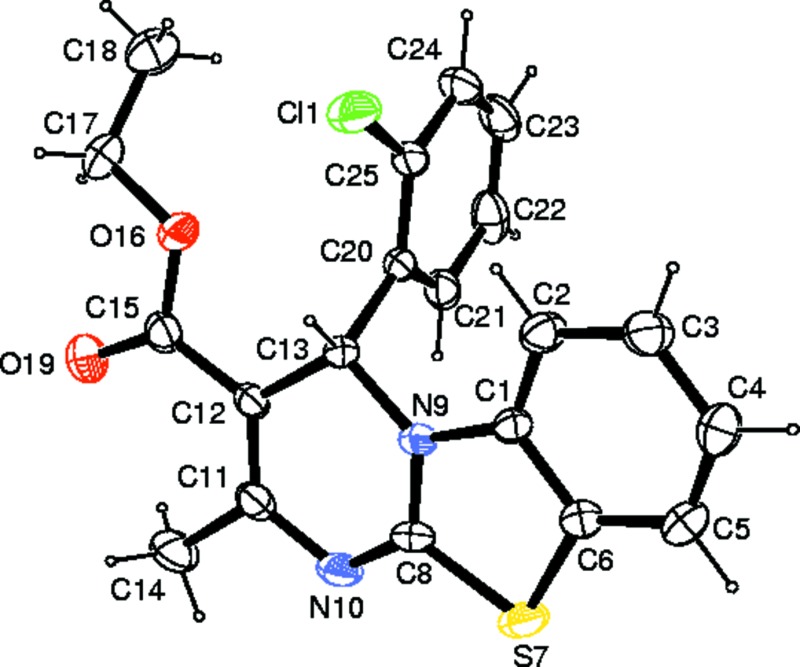
ORTEP view of the molecule with displacement ellipsoids drawn at the 40% probability level.

**Fig. 2.**
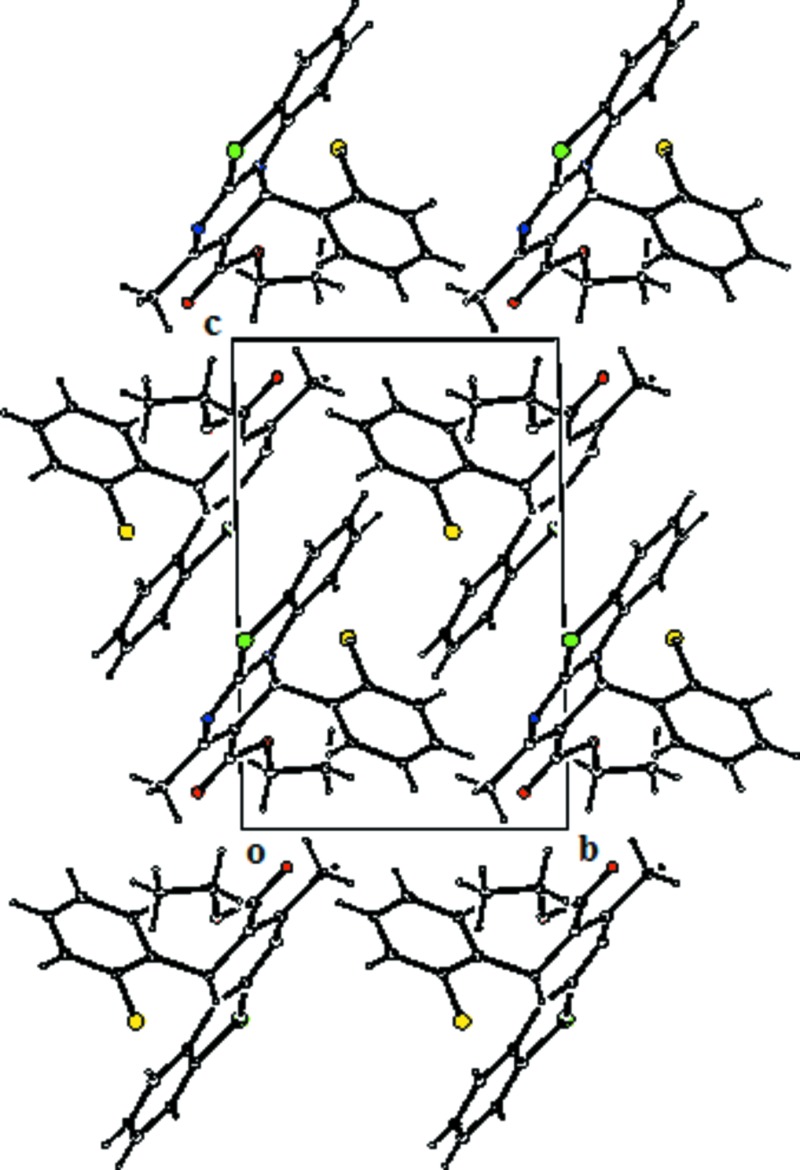
The packing arrangement of molecules viewed down the a axis.

ORTEP . DOI: 10.1107/S2056989015014905/hb7475fig1.tif
*ORTEP* view of the mol­ecule with displacement ellipsoids drawn at the 40% probability level.

a . DOI: 10.1107/S2056989015014905/hb7475fig2.tif
The packing arrangement of mol­ecules viewed down the *a* axis.

CCDC reference: 1406433

Additional supporting information:  crystallographic information; 3D view; checkCIF report

## supplementary crystallographic information

### S1. Experimental

To a mixture of ethylacetoacetate (1.0 mmol, 0.13 g), 2-chlorobenzadehyde (1.0 mmol, 0.14 g) and 2-aminobenzothiazole (1.0 mmol, 0.152 g) in a round bottom flask (25 ml), C/TiO_2_·SO_3_·SbCl_2_ (0.1 g) was added and the reaction mixture was heated at 363 K under solvent-free conditions for 1 h. Hot ethanol (2 × 5 ml) was added to the reaction mixture and the catalyst was separated by simple filteration. Removal of the solvent under reduced pressure afforded the product, which was further crystallized from ethanol as yellow crystals (Yield: 88%).

### S2. Refinement

All the H atoms were geometrically fixed and allowed to ride on their parent C atoms, with C—H distances of 0.93–0.96 Å; and with *U*_iso_(H) = 1.2*U*_eq_(C), except for the methyl group where *U*_iso_(H) = 1.5*U*_eq_(C).

### Crystal data

**Table:**

C_20_H_17_ClN_2_O_2_S	*Z* = 2
*M**_r_* = 384.87	*F*(000) = 400
Triclinic, *P*1	*D*_x_ = 1.431 Mg m^−^^3^
Hall symbol: -P 1	Mo *K*α radiation, λ = 0.71073 Å
*a* = 8.9049 (8) Å	Cell parameters from 1737 reflections
*b* = 8.9275 (10) Å	θ = 4.1–27.4°
*c* = 12.3564 (11) Å	µ = 0.35 mm^−^^1^
α = 88.434 (8)°	*T* = 293 K
β = 83.536 (7)°	Block, colourless
γ = 66.201 (10)°	0.30 × 0.20 × 0.20 mm
*V* = 892.90 (15) Å^3^	

### Data collection

**Table:**

Oxford Diffraction Xcalibur Sapphire3 diffractometer	3477 independent reflections
Radiation source: fine-focus sealed tube	2275 reflections with *I* > 2σ(*I*)
Graphite monochromator	*R*_int_ = 0.033
Detector resolution: 16.1049 pixels mm^-1^	θ_max_ = 26.0°, θ_min_ = 3.9°
ω scans	*h* = −10→10
Absorption correction: multi-scan (*CrysAlis PRO*; Oxford Diffraction, 2010)	*k* = −10→10
*T*_min_ = 0.880, *T*_max_ = 1.000	*l* = −9→15
6440 measured reflections	

### Refinement

**Table:**

Refinement on *F*^2^	Primary atom site location: structure-invariant direct methods
Least-squares matrix: full	Secondary atom site location: difference Fourier map
*R*[*F*^2^ > 2σ(*F*^2^)] = 0.053	Hydrogen site location: inferred from neighbouring sites
*wR*(*F*^2^) = 0.142	H-atom parameters constrained
*S* = 1.03	*w* = 1/[σ^2^(*F*_o_^2^) + (0.0556*P*)^2^ + 0.235*P*] where *P* = (*F*_o_^2^ + 2*F*_c_^2^)/3
3477 reflections	(Δ/σ)_max_ < 0.001
237 parameters	Δρ_max_ = 0.64 e Å^−^^3^
0 restraints	Δρ_min_ = −0.35 e Å^−^^3^

### Special details

**Table:**

Geometry. All e.s.d.'s (except the e.s.d. in the dihedral angle between two l.s. planes) are estimated using the full covariance matrix. The cell e.s.d.'s are taken into account individually in the estimation of e.s.d.'s in distances, angles and torsion angles; correlations between e.s.d.'s in cell parameters are only used when they are defined by crystal symmetry. An approximate (isotropic) treatment of cell e.s.d.'s is used for estimating e.s.d.'s involving l.s. planes.
Refinement. Refinement of *F*^2^ against ALL reflections. The weighted *R*-factor *wR* and goodness of fit *S* are based on *F*^2^, conventional *R*-factors *R* are based on *F*, with *F* set to zero for negative *F*^2^. The threshold expression of *F*^2^ > σ(*F*^2^) is used only for calculating *R*-factors(gt) *etc*. and is not relevant to the choice of reflections for refinement. *R*-factors based on *F*^2^ are statistically about twice as large as those based on *F*, and *R*- factors based on ALL data will be even larger.

### Fractional atomic coordinates and isotropic or equivalent isotropic displacement parameters (Å^2^)

**Table:**

	*x*	*y*	*z*	*U*_iso_*/*U*_eq_	
S7	0.83804 (10)	0.01946 (11)	0.38623 (8)	0.0592 (3)	
Cl1	0.02890 (10)	0.33670 (11)	0.39074 (8)	0.0648 (3)	
N9	0.5363 (3)	0.0924 (2)	0.35456 (18)	0.0370 (6)	
C25	0.1519 (4)	0.3941 (3)	0.2942 (2)	0.0431 (7)	
C12	0.4497 (3)	−0.0172 (3)	0.2052 (2)	0.0368 (7)	
O16	0.1721 (3)	0.0825 (2)	0.17927 (17)	0.0531 (6)	
N10	0.7388 (3)	−0.0979 (3)	0.2270 (2)	0.0503 (7)	
C21	0.4011 (4)	0.3408 (3)	0.1786 (2)	0.0445 (7)	
H21	0.5087	0.2715	0.1531	0.053*	
C8	0.6924 (3)	−0.0008 (3)	0.3117 (2)	0.0427 (7)	
C20	0.3121 (3)	0.2857 (3)	0.2573 (2)	0.0345 (6)	
C15	0.3219 (4)	−0.0328 (3)	0.1475 (2)	0.0446 (7)	
O19	0.3434 (3)	−0.1335 (3)	0.0766 (2)	0.0719 (7)	
C13	0.3919 (3)	0.1118 (3)	0.2977 (2)	0.0341 (6)	
H13	0.3110	0.0911	0.3495	0.041*	
C1	0.5264 (3)	0.1829 (3)	0.4491 (2)	0.0370 (7)	
C11	0.6124 (4)	−0.1115 (3)	0.1764 (2)	0.0443 (7)	
C3	0.4058 (4)	0.3661 (4)	0.6003 (3)	0.0553 (9)	
H3	0.3125	0.4372	0.6429	0.066*	
C24	0.0834 (4)	0.5496 (4)	0.2540 (3)	0.0612 (10)	
H24	−0.0238	0.6201	0.2795	0.073*	
C2	0.3873 (4)	0.2860 (4)	0.5114 (2)	0.0473 (8)	
H2	0.2829	0.3019	0.4944	0.057*	
C17	0.0396 (4)	0.0912 (4)	0.1179 (3)	0.0608 (9)	
H17A	−0.0047	0.0128	0.1455	0.073*	
H17B	0.0815	0.0642	0.0419	0.073*	
C6	0.6823 (4)	0.1580 (3)	0.4765 (2)	0.0439 (7)	
C14	0.6786 (4)	−0.2416 (4)	0.0879 (3)	0.0640 (10)	
H14A	0.6468	−0.3300	0.1087	0.096*	
H14B	0.7969	−0.2819	0.0772	0.096*	
H14C	0.6343	−0.1959	0.0214	0.096*	
C5	0.6991 (4)	0.2383 (4)	0.5662 (3)	0.0561 (9)	
H5	0.8031	0.2214	0.5845	0.067*	
C4	0.5604 (4)	0.3422 (4)	0.6264 (3)	0.0601 (9)	
H4	0.5697	0.3978	0.6861	0.072*	
C23	0.1744 (5)	0.5998 (4)	0.1761 (3)	0.0675 (11)	
H23	0.1283	0.7051	0.1491	0.081*	
C18	−0.0923 (5)	0.2586 (5)	0.1279 (4)	0.0812 (12)	
H18A	−0.1419	0.2802	0.2021	0.122*	
H18B	−0.1747	0.2677	0.0813	0.122*	
H18C	−0.0457	0.3365	0.1069	0.122*	
C22	0.3327 (5)	0.4969 (4)	0.1373 (3)	0.0594 (10)	
H22	0.3932	0.5316	0.0839	0.071*	

### Atomic displacement parameters (Å^2^)

**Table:**

	*U*^11^	*U*^22^	*U*^33^	*U*^12^	*U*^13^	*U*^23^
S7	0.0328 (4)	0.0641 (5)	0.0699 (6)	−0.0056 (4)	−0.0153 (4)	−0.0055 (5)
Cl1	0.0374 (5)	0.0726 (6)	0.0751 (6)	−0.0129 (4)	−0.0018 (4)	−0.0116 (5)
N9	0.0318 (13)	0.0316 (11)	0.0424 (14)	−0.0058 (10)	−0.0106 (11)	0.0003 (10)
C25	0.0382 (16)	0.0343 (14)	0.0534 (19)	−0.0077 (13)	−0.0169 (15)	−0.0053 (13)
C12	0.0402 (16)	0.0244 (12)	0.0425 (16)	−0.0089 (12)	−0.0075 (14)	−0.0002 (12)
O16	0.0401 (12)	0.0549 (12)	0.0632 (14)	−0.0144 (10)	−0.0165 (11)	−0.0126 (11)
N10	0.0361 (14)	0.0421 (13)	0.0579 (17)	−0.0002 (11)	−0.0040 (13)	−0.0080 (13)
C21	0.0514 (19)	0.0383 (15)	0.0456 (18)	−0.0177 (14)	−0.0141 (16)	−0.0004 (13)
C8	0.0324 (16)	0.0349 (14)	0.0517 (18)	−0.0028 (12)	−0.0107 (14)	0.0046 (14)
C20	0.0341 (15)	0.0295 (13)	0.0395 (16)	−0.0100 (12)	−0.0131 (13)	−0.0019 (12)
C15	0.0512 (19)	0.0349 (15)	0.0499 (18)	−0.0189 (14)	−0.0085 (16)	0.0017 (14)
O19	0.0711 (17)	0.0584 (14)	0.0851 (18)	−0.0210 (13)	−0.0161 (15)	−0.0285 (13)
C13	0.0299 (14)	0.0297 (13)	0.0421 (16)	−0.0097 (11)	−0.0104 (13)	0.0026 (12)
C1	0.0351 (16)	0.0344 (14)	0.0413 (16)	−0.0112 (12)	−0.0152 (13)	0.0058 (13)
C11	0.0492 (18)	0.0291 (14)	0.0487 (18)	−0.0092 (13)	−0.0068 (15)	−0.0011 (13)
C3	0.050 (2)	0.0590 (19)	0.0467 (19)	−0.0105 (16)	−0.0080 (16)	−0.0088 (16)
C24	0.053 (2)	0.0373 (17)	0.081 (3)	0.0000 (16)	−0.028 (2)	−0.0075 (17)
C2	0.0403 (17)	0.0554 (18)	0.0430 (17)	−0.0143 (15)	−0.0111 (15)	−0.0004 (15)
C17	0.052 (2)	0.068 (2)	0.074 (2)	−0.0304 (18)	−0.0224 (19)	−0.0030 (18)
C6	0.0400 (17)	0.0410 (15)	0.0484 (18)	−0.0121 (13)	−0.0129 (15)	0.0037 (14)
C14	0.058 (2)	0.0497 (18)	0.068 (2)	−0.0050 (17)	−0.0011 (19)	−0.0180 (18)
C5	0.048 (2)	0.064 (2)	0.060 (2)	−0.0208 (17)	−0.0246 (18)	0.0024 (18)
C4	0.065 (2)	0.066 (2)	0.053 (2)	−0.0261 (19)	−0.0207 (19)	−0.0087 (18)
C23	0.087 (3)	0.0312 (16)	0.079 (3)	−0.0107 (19)	−0.042 (2)	0.0046 (18)
C18	0.064 (3)	0.082 (3)	0.098 (3)	−0.022 (2)	−0.043 (2)	0.008 (2)
C22	0.092 (3)	0.0491 (19)	0.051 (2)	−0.039 (2)	−0.021 (2)	0.0095 (16)

### Geometric parameters (Å, º)

**Table:**

S7—C8	1.741 (3)	C11—C14	1.505 (4)
S7—C6	1.744 (3)	C3—C4	1.379 (4)
Cl1—C25	1.735 (3)	C3—C2	1.388 (4)
N9—C8	1.353 (3)	C3—H3	0.9300
N9—C1	1.413 (3)	C24—C23	1.370 (5)
N9—C13	1.481 (3)	C24—H24	0.9300
C25—C24	1.376 (4)	C2—H2	0.9300
C25—C20	1.394 (4)	C17—C18	1.479 (5)
C12—C11	1.360 (4)	C17—H17A	0.9700
C12—C15	1.462 (4)	C17—H17B	0.9700
C12—C13	1.536 (3)	C6—C5	1.390 (4)
O16—C15	1.338 (3)	C14—H14A	0.9600
O16—C17	1.448 (3)	C14—H14B	0.9600
N10—C8	1.296 (4)	C14—H14C	0.9600
N10—C11	1.394 (4)	C5—C4	1.359 (4)
C21—C22	1.385 (4)	C5—H5	0.9300
C21—C20	1.389 (4)	C4—H4	0.9300
C21—H21	0.9300	C23—C22	1.374 (5)
C20—C13	1.521 (3)	C23—H23	0.9300
C15—O19	1.216 (3)	C18—H18A	0.9600
C13—H13	0.9800	C18—H18B	0.9600
C1—C2	1.370 (4)	C18—H18C	0.9600
C1—C6	1.394 (4)	C22—H22	0.9300
			
C8—S7—C6	91.01 (14)	C23—C24—C25	119.5 (3)
C8—N9—C1	114.1 (2)	C23—C24—H24	120.2
C8—N9—C13	121.6 (2)	C25—C24—H24	120.2
C1—N9—C13	123.8 (2)	C1—C2—C3	118.5 (3)
C24—C25—C20	121.5 (3)	C1—C2—H2	120.7
C24—C25—Cl1	117.2 (3)	C3—C2—H2	120.7
C20—C25—Cl1	121.3 (2)	O16—C17—C18	109.4 (3)
C11—C12—C15	121.0 (2)	O16—C17—H17A	109.8
C11—C12—C13	121.9 (2)	C18—C17—H17A	109.8
C15—C12—C13	117.1 (2)	O16—C17—H17B	109.8
C15—O16—C17	116.6 (2)	C18—C17—H17B	109.8
C8—N10—C11	115.9 (2)	H17A—C17—H17B	108.2
C22—C21—C20	121.3 (3)	C5—C6—C1	120.7 (3)
C22—C21—H21	119.3	C5—C6—S7	128.0 (2)
C20—C21—H21	119.3	C1—C6—S7	111.2 (2)
N10—C8—N9	127.7 (3)	C11—C14—H14A	109.5
N10—C8—S7	120.5 (2)	C11—C14—H14B	109.5
N9—C8—S7	111.8 (2)	H14A—C14—H14B	109.5
C21—C20—C25	117.5 (3)	C11—C14—H14C	109.5
C21—C20—C13	119.1 (2)	H14A—C14—H14C	109.5
C25—C20—C13	123.4 (3)	H14B—C14—H14C	109.5
O19—C15—O16	121.7 (3)	C4—C5—C6	118.6 (3)
O19—C15—C12	126.3 (3)	C4—C5—H5	120.7
O16—C15—C12	112.0 (2)	C6—C5—H5	120.7
N9—C13—C20	110.1 (2)	C5—C4—C3	120.9 (3)
N9—C13—C12	108.4 (2)	C5—C4—H4	119.6
C20—C13—C12	112.7 (2)	C3—C4—H4	119.6
N9—C13—H13	108.5	C24—C23—C22	120.9 (3)
C20—C13—H13	108.5	C24—C23—H23	119.5
C12—C13—H13	108.5	C22—C23—H23	119.5
C2—C1—C6	120.2 (2)	C17—C18—H18A	109.5
C2—C1—N9	128.0 (2)	C17—C18—H18B	109.5
C6—C1—N9	111.9 (2)	H18A—C18—H18B	109.5
C12—C11—N10	123.1 (2)	C17—C18—H18C	109.5
C12—C11—C14	125.1 (3)	H18A—C18—H18C	109.5
N10—C11—C14	111.8 (3)	H18B—C18—H18C	109.5
C4—C3—C2	121.1 (3)	C23—C22—C21	119.2 (4)
C4—C3—H3	119.5	C23—C22—H22	120.4
C2—C3—H3	119.5	C21—C22—H22	120.4
			
C11—N10—C8—N9	−2.2 (5)	C15—C12—C13—C20	−65.5 (3)
C11—N10—C8—S7	176.9 (2)	C8—N9—C1—C2	−179.2 (3)
C1—N9—C8—N10	178.7 (3)	C13—N9—C1—C2	8.4 (4)
C13—N9—C8—N10	−8.7 (5)	C8—N9—C1—C6	1.1 (3)
C1—N9—C8—S7	−0.4 (3)	C13—N9—C1—C6	−171.3 (2)
C13—N9—C8—S7	172.18 (18)	C15—C12—C11—N10	178.2 (3)
C6—S7—C8—N10	−179.5 (3)	C13—C12—C11—N10	−0.7 (4)
C6—S7—C8—N9	−0.3 (2)	C15—C12—C11—C14	−2.0 (5)
C22—C21—C20—C25	−0.7 (4)	C13—C12—C11—C14	179.1 (3)
C22—C21—C20—C13	178.5 (2)	C8—N10—C11—C12	6.8 (4)
C24—C25—C20—C21	0.4 (4)	C8—N10—C11—C14	−173.1 (3)
Cl1—C25—C20—C21	178.98 (19)	C20—C25—C24—C23	−0.2 (4)
C24—C25—C20—C13	−178.9 (2)	Cl1—C25—C24—C23	−178.8 (2)
Cl1—C25—C20—C13	−0.3 (4)	C6—C1—C2—C3	1.1 (4)
C17—O16—C15—O19	−5.8 (4)	N9—C1—C2—C3	−178.6 (3)
C17—O16—C15—C12	172.5 (2)	C4—C3—C2—C1	−0.5 (5)
C11—C12—C15—O19	5.4 (5)	C15—O16—C17—C18	−155.2 (3)
C13—C12—C15—O19	−175.7 (3)	C2—C1—C6—C5	−0.8 (4)
C11—C12—C15—O16	−172.8 (3)	N9—C1—C6—C5	179.0 (3)
C13—C12—C15—O16	6.1 (4)	C2—C1—C6—S7	179.0 (2)
C8—N9—C13—C20	−110.7 (3)	N9—C1—C6—S7	−1.3 (3)
C1—N9—C13—C20	61.2 (3)	C8—S7—C6—C5	−179.4 (3)
C8—N9—C13—C12	12.9 (3)	C8—S7—C6—C1	0.9 (2)
C1—N9—C13—C12	−175.2 (2)	C1—C6—C5—C4	−0.2 (5)
C21—C20—C13—N9	64.4 (3)	S7—C6—C5—C4	−179.9 (3)
C25—C20—C13—N9	−116.4 (3)	C6—C5—C4—C3	0.8 (5)
C21—C20—C13—C12	−56.7 (3)	C2—C3—C4—C5	−0.5 (5)
C25—C20—C13—C12	122.5 (3)	C25—C24—C23—C22	0.3 (5)
C11—C12—C13—N9	−8.7 (4)	C24—C23—C22—C21	−0.6 (5)
C15—C12—C13—N9	172.4 (2)	C20—C21—C22—C23	0.9 (4)
C11—C12—C13—C20	113.4 (3)		
