# Supplementary figures and images for: Crystal structure of ethyl 4-(2-chloro­phen­yl)-2-methyl-4H-pyrimido[2,1-b][1,3]benzo­thia­zole-3-carboxyl­ate
Source: Acta Crystallogr E Crystallogr Commun. 2015 Aug 22;71(Pt 9):o669. doi: 10.1107/S2056989015014905 (PMC4555405; doi:10.1107/S2056989015014905)

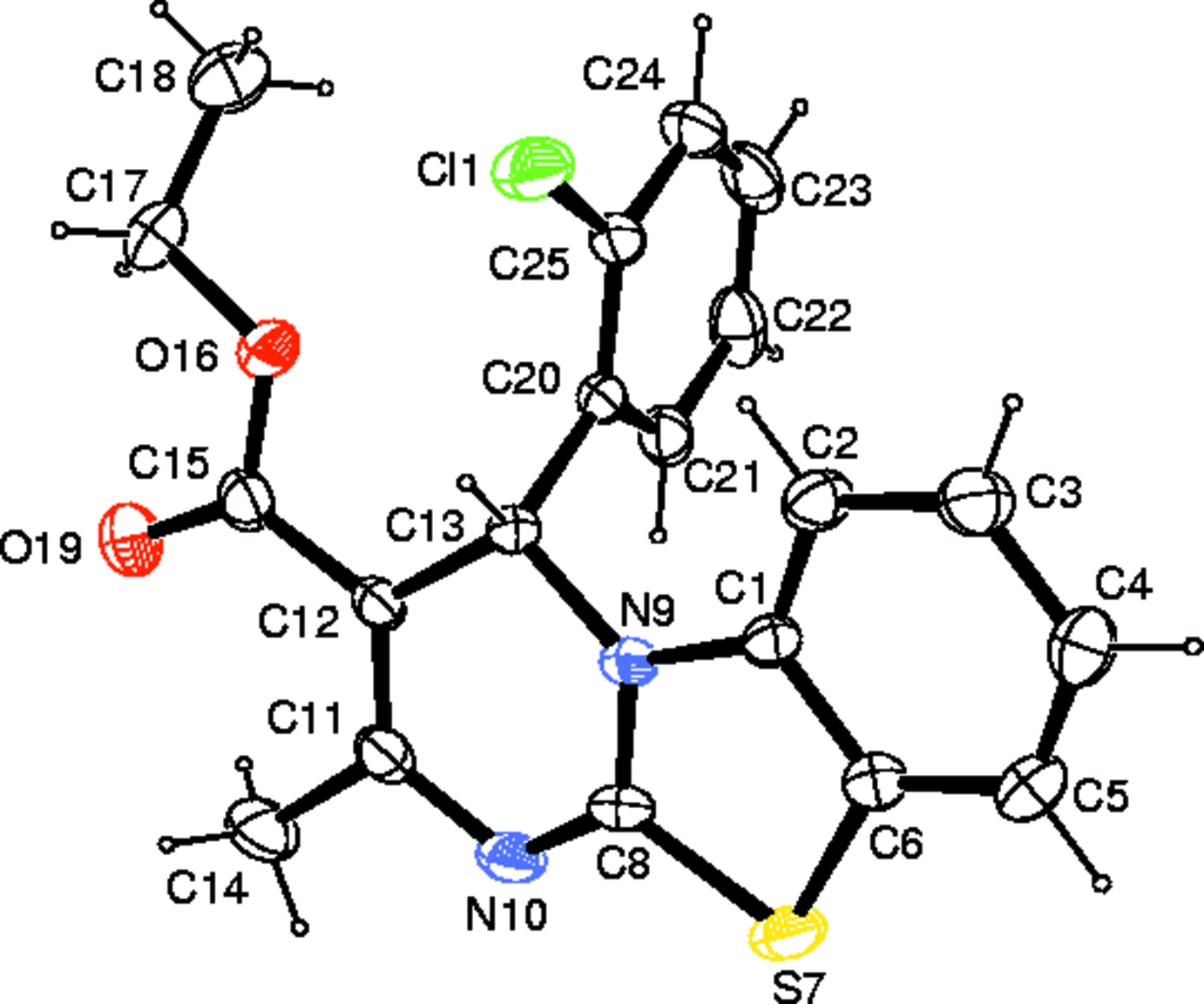

Supplement: Supplementary file 4 [file e-71-0o669-fig1.tif]

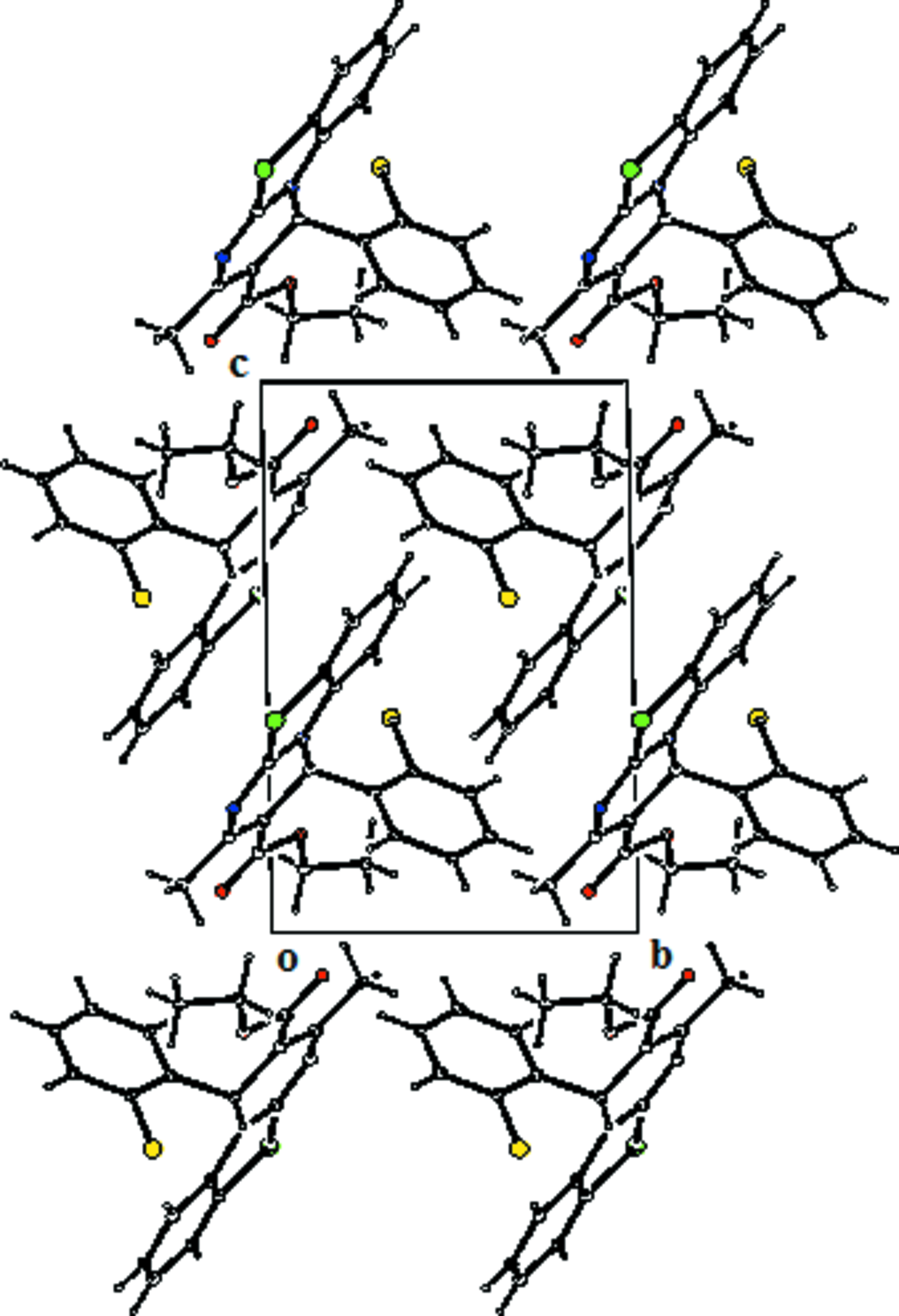

Supplement: Supplementary file 5 [file e-71-0o669-fig2.tif]
